# Pre- and post-shunting observations in adult sheep with kaolin-induced hydrocephalus

**DOI:** 10.1186/2045-8118-10-24

**Published:** 2013-07-11

**Authors:** Miles G Johnston, Marc R Del Bigio, James M Drake, Dianna Armstrong, Domenico L Di Curzio, Jeff Bertrand

**Affiliations:** 1 Brain Sciences, Sunnybrook Research Institute and Department of Laboratory Medicine and Pathobiology, University of Toronto, 2075 Bayview Avenue, Toronto, ON M4N 3M5, Canada; 2 Department of Pathology, University of Manitoba, Winnipeg, MB, Canada; 3 Department of Neurosurgery, Hospital for Sick Children, Toronto, ON, Canada; 4 Manitoba Institute of Child Health, Winnipeg, MB, Canada; 5 Medtronic Neurosurgery, Goleta, CA, USA

**Keywords:** Hydrocephalus, Shunting, Animal model

## Abstract

**Background:**

The objective of this study was to examine host-shunt interactions in sheep with kaolin-induced hydrocephalus.

**Methods:**

Forty-two sheep (29–40 kg) were utilized for this study. In 20 animals, various kaolin doses were injected into the cisterna magna including 10 and 50 mg/kg as well as 2–4 ml of a 25% kaolin suspension. Based on animal health and hydrocephalus development, 3 ml of a 25% kaolin suspension was chosen. In 16 animals, kaolin was administered and 6–8 days later, the animals received a custom made ventriculo-peritoneal shunt. In 8 animals ventricular CSF pressures were measured with a water manometer before kaolin administration and 7–8 days later. The sheep were allowed to survive for up to 9–12 weeks post-kaolin or until clinical status required euthanasia. Brains were assessed for morphological and histological changes. Ventricle/cerebrum cross sectional area ratios (V/C) were calculated from photographs of the sliced coronal planes immediately anterior to the interventricular foramina.

**Results:**

Intraventricular pressures increased from 12.4±1.1 cm H_2_O to 41.3±3.5 cm H_2_O following kaolin injection (*p* < 0.0001, n = 8). In all animals, we observed kaolin on the basal surface of the brain and mild (V/C 0.03-0.10) to moderate (V/C >0.10) ventricular expansion. The animals lost weight between kaolin administration and shunting (33.7±1.2 kg versus 31.0±1.7 kg) with weights after shunting remaining stable up to sacrifice (31.6±2.2 kg). Of 16 shunted animals, 5 did well and were sacrificed 9–12 weeks post-kaolin. In the remainder, the study was terminated at various times due to deteriorating health. Hydrocephalus was associated with thinning of the corpus callosum, but no obvious loss of myelin staining, along with reactive astroglial (glial fibrillary acidic immunoreactive) and microglial (Iba1 immunoreactive) changes in the white matter. Ventricular shunts revealed choroid plexus ingrowth in 5/16, brain tissue ingrowth in 1/16, problems with shunt insertion in 3/16, occlusion by hemorrhagic-inflammatory material in 5/16, or no obstruction in 2/16. Free flowing CSF indicated that the peritoneal catheter was patent.

**Conclusions:**

Cerebrospinal fluid shunts in hydrocephalic sheep fail in ways that are reminiscent of human neurosurgical experience suggesting that this model may be helpful in the development of more effective shunt technology.

## Introduction

The diversion of cerebrospinal fluid (CSF) with shunts has proven to be an effective but problematic method to treat hydrocephalus [1]. Unfortunately, about 40% of the shunts used in the treatment of pediatric hydrocephalus fail within the first year [2]. Indeed, newly diagnosed hydrocephalic patients can expect to undergo 2–4 insertions or revisions of shunts in the first 10 years after diagnosis [3]. With this in mind, many improvements have been made to shunt design over the last 50 years, but patient outcomes with the new devices have not improved to the extent one might have predicted [2,4].

Shunt failure can occur anywhere along the shunt system but most commonly, occlusion occurs at the proximal (ventricular) end. Apart from the possibility of shunt misplacement or disconnection, obstruction is often due to the ingrowth of choroid plexus into the proximal catheter but hemorrhage, infection, inflammatory debris and many other factors can also be involved [5,6]. Perhaps surprisingly, the various shunt designs have largely failed to negate these issues and shunt blockage continues to be a problem [2].

The objective of this study was to examine host-shunt interactions in an animal model of kaolin-induced hydrocephalus. To provide a realistic shunt environment, a larger species would allow shunt size and design similar to its human counterpart. The use of sheep is appropriate because much is known of its CSF physiology [7-10]*.* Additionally, kaolin has been used to induce hydrocephalus in fetal sheep [11,12] and has also been used to create syringomyelia in adult sheep [13]. As part of this study, the relationship between shunt success/failure with the clinical status was assessed. We report that the causes of shunt malfunction in this model mirrored those observed in clinical practice.

## Materials and methods

Forty-three randomly bred sheep (*Ovis aries*, Dorset breed) weighing 29–40 kg were used for this investigation. In one group (20 animals), we assessed the effects of various kaolin doses on ventricle size and animal health. In a second group (5 animals), we determined the shunting protocol. In the final group (16 sheep), the animals received kaolin followed by surgery for a ventriculo-peritoneal shunt. Two normal sheep served as controls for ventricle size assessment. They were fed hay, pellets and water ad libitum, but were fasted 24 h before surgery. Experiments were approved by the ethics committee at Sunnybrook Health Sciences Centre, and conformed to the guidelines set by the Canadian Council on Animal Care and the Animals for Research Act of Ontario.

### Animal preparation

The sheep were admitted to the animal facility one week prior to the study for acclimatization and health checks. All animals received an i.m. or s.c. injection of Tribrissen 48% (trimethoprim and sulfadiazine) one day prior to surgery and daily for 4 days post surgery.

To induce anesthesia, sodium thiopental was administered i.v. via the cephalic or jugular vein (15-25 ml/kg). Following this, the animals were intubated with a Sheridan/HVT tracheal tube and ventilated with 2-3% isoflurane in O_2_ using a respirator (Hallowell 2000, DRE Veterinary, Louisville, USA). Thirty minutes before surgery, the sheep were infused with 1 g of cefazolin i.v. in 100 ml of saline over a 30-min time period. Each animal also received an i.m. injection of temgesic (buprenorphine 0.005 mg/kg). Later animals received a duragesic (fentanyl) patch (50 mg) pre-operatively, which was replaced 3 days after surgery if required. We found that the combination of buprenorphine and fentanyl kept the animals more comfortable post operatively.

### Access to cisterna magna and injection of kaolin

The surgical site was shaved, cleaned and prepped with a 70% alcohol and povidone-iodine scrub. Under sterile surgical technique, a skin incision was made in the neck caudal to the exterior occipital protuberance. The fibers of the ligamentum nuchae were separated in the midline as well as the underlying muscle layers. The dissection was carried out until the occipital-axial ligament was exposed. An 18 or 20 Gauge angiocatheter was inserted into the subarachnoid space. The sterile kaolin (aluminum silicate) suspension was injected manually over 5 min into the cisterna magna. Prior to the kaolin injection, the same volume of CSF was withdrawn. The muscle was then sutured using 2–0 absorbable (polyglycolic acid, Dexon) continuous stitches and the skin was closed with 2–0 silk interrupted stitches. The wound was sprayed with a topical analgesic/anti bacterial spray (Boroform, Sanofi-Aventis, Laval, Canada).

Almost all of the animals decreased their water consumption following the surgery. Consequently, Ringers lactate (1 L) was given i.v. 3 days post operatively. Post surgical monitoring was maintained until the animal was fully recovered. Analgesics were given as necessary. Animals were examined frequently for the behavioral signs of elevated ICP including loss of appetite, nasal discharge, grinding teeth, drooping ears, lowered head, the inability to stand and pawing at the ground with the forelimbs. Any animals that stopped eating, exhibited head butting or remained in sternal recumbency and were not getting relief from analgesics were deemed to be in significant distress and were euthanized.

### Shunting procedure

A ventriculo-peritoneal shunt was installed between the 6th and 8th day post-kaolin injection. These were custom made at Medtronic Corporation (Goleta CA) and the parts were of the same materials and quality as those used for human shunts. Standard production “Ultra Small” valves with a Low pressure rating (cat# 22017 B-LL) were utilized in this application to ensure that the shunts would flow in the more horizontal orientation of the sheep vs. human. Standard- sized tubing 2.54 mm OD and 1.27 mm ID was employed and the ventricular catheters (4 cm in length) were modified slit tip InnerVision type with the flow-hole pattern shortened to accommodate the smaller ventricles in the sheep. The flow-hole size was the standard 0.46 mm diameter. The length of tubing between the snap reservoir and the valve was lengthened slightly to better accommodate the sheep anatomy and the distal catheter was set at 120 cm in length. Shunts were pre-soaked in a solution containing bacitracin (50,000 units) in 30 ml NaCl.

The surgical site was shaved, cleaned and prepped with an alcohol and povidone-iodine scrub and the head secured in a custom built frame. Under sterile surgical techniques, a 3 cm incision was made in the skin on the scalp in the right parietal-occipital area. A hole was drilled 2 cm off of the midline, just above the lambdoid suture with a midline trajectory. The ventricular shunt catheter was then inserted into the lateral ventricle of the brain. The correct position was confirmed by the outflow of CSF. In some animals, a water manometer system was used to determine the intracranial pressure (ICP). A peritoneal incision was made 5 cm below the left rib cage. The outflow catheter of the shunt was tunneled subcutaneously using a Medtronic tunneller and the distal end was inserted into the peritoneal cavity. Once CSF flow had been confirmed, the distal catheter was anchored to the subcutaneous tissue, muscle and skin with 2–0 silk sutures. A topical spray, boroform was applied to the wounds until fully healed. The sutures were removed 7 to 10 days post surgery. As before, the animals were examined frequently and those in severe stress were euthanized.

### Measurements of CSF pressure

In eight animals, CSF pressure was measured just before kaolin injection and approximately 1 week later, either before shunt implantation (4 animals) or in the non-shunted group (4 animals). In the first case, pressures were measured from the angiocatheter that was inserted into the cisterna magna. Immediately prior to Kaolin injection, an arterial line (Hospira, San Jose, USA) was attached to the angiocatheter. The distal portion of the line was raised and CSF pressure was measured using a water post manometer. One week later, before shunt placement, the lateral ventricle was punctured with a 14 GA needle and CSF pressure was determined as above. The needle was replaced with the intraventricular catheter. Data are expressed as mean **±** SΕΜ**.** Pressures pre- and post-kaolin were analysed using the paired Students t-test.

### Experimental protocols

Initial studies (10 animals) were designed to test how the animals responded to kaolin injection into the cisterna magna and to gain experience with shunt implantation. We attempted injections of 10 or 50 mg/kg in 2.5 ml volumes or administration of kaolin as a 25% suspension (in saline) in volumes between 1 and 4 ml. These animals were sacrificed at various times. Based on animal health and hydrocephalus development, injection of 3 ml of a 25% kaolin suspension appeared to be the most appropriate dose for further studies. In addition, shunts were placed in 5 sheep to optimize the shunting protocol. From this point on, the main study commenced.

Two groups of studies were performed. In the first, we addressed the natural history of kaolin-induced hydrocephalus in un-shunted animals (10 sheep). These animals were allowed to survive up to 3 weeks depending on their clinical status. We monitored their status until it was clear that they must be sacrificed or until their clinical status suggested that they had recovered from the insult. At the appropriate time as defined above, they were sacrificed. In the second group, we examined the impact of shunting on the clinical status of kaolin-injected animals (16 sheep). These animals were shunted after 1 week. They were then monitored for up to 4 months or until their clinical status suggested that they must be sacrificed.

### Morphological and histological analysis

On the day of sacrifice, the animal was anesthetized as described earlier. The distal portion of the shunt was removed from the peritoneal cavity to confirm CSF outflow. The incision on the scalp was opened and the drainage catheter was disconnected from the ventricular catheter. The animal was then sacrificed with 20 ml of euthanol (pentobarbital 240 mg/ml) given i.v. The brain was removed and visual observation of kaolin distribution was made at this point. The brain was immersion fixed in 10% buffered formalin, and transferred to 100% alcohol 1 week later for shipment to the pathology lab. The neuropathologist was blinded to the timing of the kaolin injection and shunt duration.

The fixed brain was photographed then sectioned in the coronal plane at approximately 5 mm intervals. Location of the shunt catheter was verified during sectioning and all sections were photographed. Samples for histological examination included: a) the frontal lobe including the corpus callosum and periventricular region, b) tissue adherent to the shunt catheter tip, c) the hippocampus opposite the shunt entry point, d) brain surrounding the shunt catheter entry site, e) midbrain, f) medulla oblongata and cerebellum. These tissues were dehydrated in alcohol, embedded in paraffin, sectioned at 5 μm, and stained with hematoxylin and eosin (H&E). Selected sections were stained with solochrome cyanin (to demonstrate myelin), Gram stain (to demonstrate bacteria) or Masson trichrome stain (to demonstrate collagen). Selected frontal sections were immunostained to detect reactive astrocytes (polyclonal rabbit glial fibrillary acidic protein - GFAP, 1/15000 dilution; DAKO Z0334; Glostrup, Denmark) and activated microglia (polyclonal rabbit Iba-1, 1/5000 dilution; Synaptic Systems 234 003; Gottingen, Germany). Antigen retrieval was used to enhance labeling of Iba-1 by microwaving slides in 0.01 M sodium citrate buffer pH 6.0 for 20 min. Primary antibodies underwent 1 h incubation at room temperature followed by incubation with appropriate biotinylated anti-rabbit IgG antibody, followed by reaction with streptavidin-peroxidase, detection with diaminobenzidine (DAB, Sigma D5905, St. Louis, USA), and finally counterstaining with hematoxylin. Negative controls were processed without the primary antibody.

### Assessment of ventricle size

An analysis was made of the ventricle/cerebrum cross sectional area ratios (V/C) calculated from photographs of the brains. The images were obtained from the sliced coronal planes including the frontal horns of the lateral ventricles on the slice immediately anterior to the interventricular foramena. The tracings were assessed using ImageJ software. Based on our experience in sheep and the data from the non-treated control animal it would appear that V/C ratios of 0.03 and under are normal. We somewhat arbitrarily concluded that ratios between 0.03 and 0.1 indicated mild ventricular expansion and ratios over 0.1 were classified as moderate hydrocephalus.

## Results

### Appropriate kaolin dose

We approached this issue from two perspectives; injecting a mass of kaolin based on animal weight and administering kaolin as a 25% suspension in different volumes. In the first case (mass/weight), 50 mg/kg was toxic. The animals receiving 10 ml/kg fared better but none of the sheep in the 50 or 10 ml/kg group developed hydrocephalus. In contrast, the animals that received 2, 3 or 4 ml of the 25% kaolin suspension exhibited mild to moderate degrees of ventriculomegaly. Modest ventricular dilation is probably the best that can be achieved in adult animals. In animals that were shunted without kaolin administration, the animals tolerated the procedures well and were sacrificed 9 weeks after shunt implantation with no complications. We used 3 ml of a 25% suspension for all subsequent studies.

### Clinical status of animals that received 3 ml of 25% kaolin suspension – no shunting

The injection of kaolin had a significant negative impact on the animal’s health and as a consequence, the sheep required constant care. One of the most serious consequences was the reluctance of the animals to drink water. This necessitated the administration of water *via* oral gavage or intravenous means. As the first week progressed, in almost all cases, the animal’s condition deteriorated markedly. This was manifested by lowered head, grinding teeth, lip curl, refusing to stand, fore leg stiffening and weakness, and occasionally convulsions. Out of the 10 animals in this group, 5 were euthanized 7 days after kaolin injection; the others were kept until their clinical condition required termination. The body weights declined from an average of 35.0 ± 1.1 kg pre-kaolin to 32.9 ± 0.7 kg at time of sacrifice.

### CSF pressure

Eight sheep had pressure measurement prior to kaolin injection, four had pressure monitoring at the time of sacrifice on day 7, and four others subsequently were shunted. Resting pressures averaged 12.4 ± 1.1 cm H_2_O. Pressures increased significantly at 7–8 days post-kaolin averaging 41.3 ± 3.5 cm H_2_O (*p* < 0.0001, n = 8, paired t test). In 5 sheep, we measured CSF pressure from the ventricles and the lumbar subarachnoid space. In every case, the spinal pressures were much less than those in the ventricles averaging 13.2 ± 1.7 cm H_2_O.

### Clinical status of animals post-shunting

With one exception, the animals were shunted 6–8 days after kaolin injection. The status of the animals after shunt treatment was quite variable, although many of the sheep improved initially and were eating and drinking normally. In 5 cases, the condition of the sheep improved and they remained stable until the decision was made to terminate the experiment. However, in the 11 other cases, the clinical condition eventually deteriorated. Evidence of declining health included loss of appetite, nasal discharge, drooping ears, lowered head, the inability to stand, pawing at the ground with the forelimbs, elevated temperatures and poor coordination. At this point, the sheep were euthanized. The time to sacrifice was variable but clinical considerations required sacrifice on average 7 days after shunt treatment.

The animals lost weight between kaolin administration and shunting (33.7 ± 1.2 kg versus 31.0 ± 1.7 kg). On sacrifice, body weights were close to pre-kaolin levels (31.6 ± 2.2 kg). In 3 of the animals that were stable, weights actually increased (in one case by 8 kg above the pre-shunt weight).

### Assessment of hydrocephalus in the animals receiving the 25% kaolin suspension – non shunted

**Figure 1:**
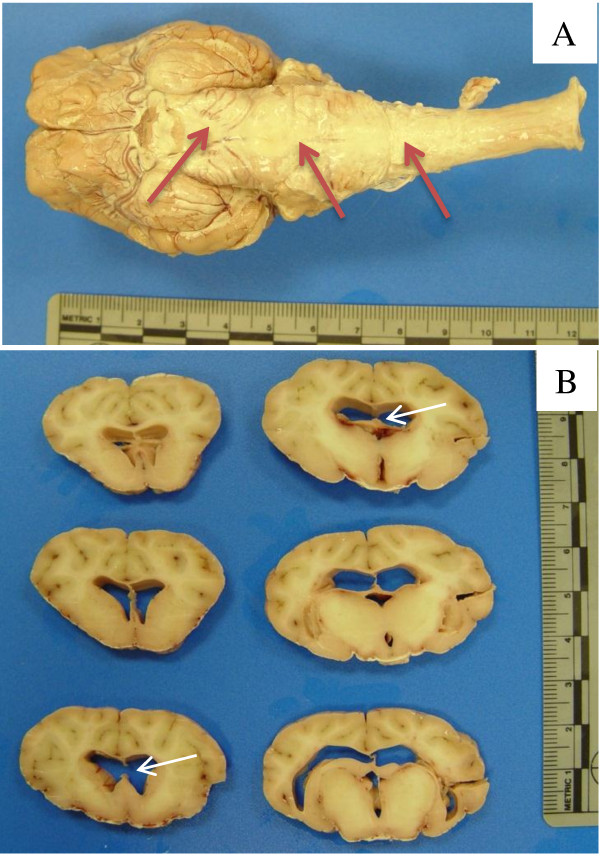
**Brain from non-shunted sheep 497P that received 3 ml of a 25% suspension of kaolin. A)** Image showing distribution of kaolin (red arrows) on the basal surface of the brain. **B)** Coronal sections through the cerebral hemispheres demonstrate mild symmetric enlargement of the lateral ventricles (V/C ratio of 0.082) and disruption of the septum pellucidum (white arrows).

Ten non-shunted sheep were examined after kaolin injection (five on day 7, one on day 12, four on days 21–23). Data for these animals are provided in Table 1. Kaolin appeared as a yellowish white deposit surrounding the medulla and upper cervical spinal cord, near the cerebellum outlets, on the basal surface of the pons and midbrain, surrounding the infundibulum, on the inferior surface of the temporal lobes including around the middle cerebral arteries (example illustrated in Figure 1A), and in some cases extending forward towards the olfactory bulbs.

**Table 1:** Ventricular dilatation in hydrocephalic sheep not treated with shunts

**Sheep #**	**Post-kaolin**	**Ventricle**
	**Day of sacrifice**	**Enlargement**
		**V/C**
391P	7	Mild
		(0.078)
461P	21	Mild
		(0.049)
001	21	Mild
		(0.066)
216R	7	Mild
		(0.056)
239R	7	Mild
		(0.066)
274R	7	Mild
		(0.063)
245R	7	Mild
		(0.050)
461P	21	Mild
		(0.049)
497P	12	Mild
		(0.082)
501P	23	Moderate
		(0.116)

Based on the post-mortem analysis of the brains, the ratio of the frontal ventricle to cerebrum cross sectional area in the control sheep was 0.029 ± .001 (mean ± SEM), in the 7–12 day hydrocephalics 0.066 ± 0.005, and in the 21–23 day hydrocephalics 0.077 ± .017. Owing to the small number of normal sheep (n = 2) these differences only approach statistical significance (*p* = 0.07, one-tailed Student t test). The enlargement of the lateral ventricles including the frontal and temporal horns (Table 1; Figure 1B) was symmetric and in a few cases, moderate expansion of the fourth ventricle was observed. The state of the cerebral aqueduct was variable with mild widening in most animals.

**Figure 2:**
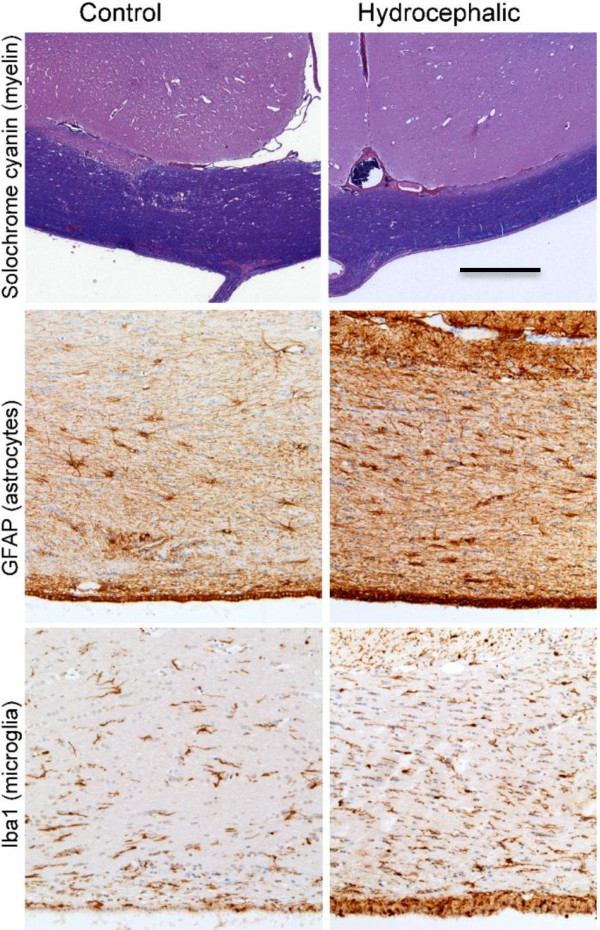
**Photomicrographs showing changes in the periventricular white matter of hydrocephalic sheep compared to intact controls.** There is thinning of the corpus callosum (solochrome cyanin stain for myelin), an increase in reactive astrocytes (GFAP), and an increase in reactive microglia (Iba1). Bar = 500 μm for top row and 100 μm for middle and bottom row.

Histologic examination of the subarachnoid compartment showed a mixed inflammatory process, predominantly macrophages (filled with kaolin particles), lymphocytes, and a few neutrophils. . Some kaolin was present within macrophages located in the choroid plexus of the fourth ventricle. There were no abnormalities, including no inflammation, in the brain tissue adjacent to the kaolin deposits. Ventricular enlargement was not associated with abnormalities in the cerebral cortex, hippocampus, or periventricular gray matter structures (striatum, thalamus). There was mild thinning and focal loss of the ependymal layer of the frontal horns. The corpus callosum was elevated and slightly thinner than in controls but there was no loss of myelin staining. Immunostaining for GFAP showed reactive astrocytes in the periventricular white matter and in the cerebral cortex. The cell bodies were not hypertrophic. Immunostaining for Iba1 showed reactive microglia only in the periventricular white matter. These cells had extensive processes but the cell bodies were not engorged (Figure 2).

### Analysis of brains in shunted group

**Figure 3:**
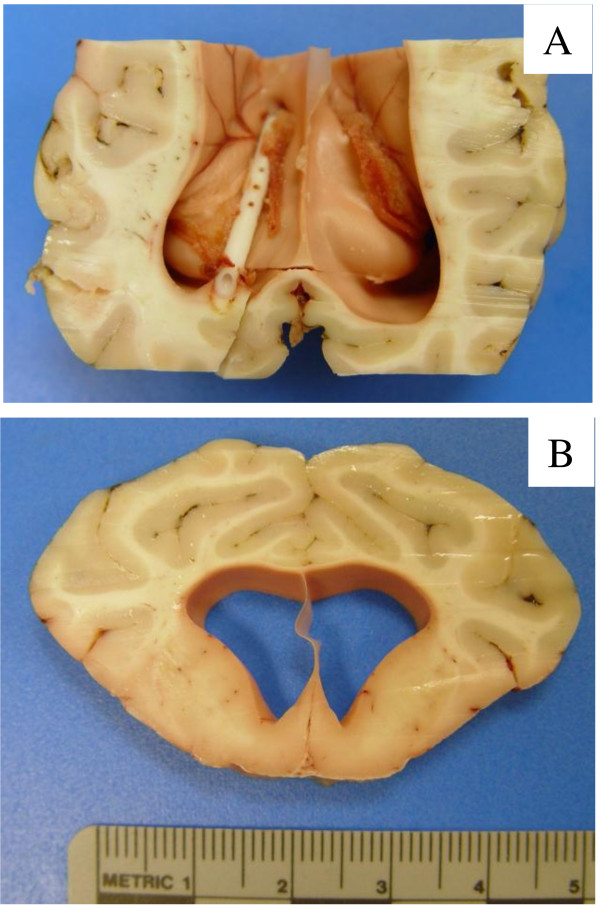
**Images of brain from shunted sheep. A)** Axial section of brain from shunted sheep 77R demonstrating enlarged ventricles and the choroid plexus surrounding the catheter. This animal was found dead in its cage with shunt occlusion 16 days after kaolin injection and 9 days after shunt placement. **B)** Coronal section of brain from shunted sheep 77R. The lateral ventricles are moderately enlarged (V/C ratio of 0.180) with elevation and thinning of the corpus callosum.

With the exception of 1 sheep that did well after shunting and demonstrated normal sized ventricles, on sacrifice all other animals demonstrated mild (8 animals) to moderate degrees of hydrocephalus (5 animals; see Table 2 for summary). The central canal of the spinal cord was mildly enlarged in 2 sheep. An example of moderate hydrocephalus in this group is illustrated in Figure 3B. In 2 sheep, the shunt surgery was unsuccessful with problems related to shunt insertion in 1 and intraoperative hemorrhage in the other. On post-mortem examination of the 14 remaining animals, shunt malfunction appeared to be due to choroid plexus ingrowth in 5 animals (36%), brain tissue ingrowth in 1 (7%), misplacement of shunt in 2 (14%), and occlusion by hemorrhagic or inflammatory material in 4 (29%). Two sheep (14%) had apparently successful shunts with no obstruction although the ventricles remained slightly enlarged. The catheter tract through the occipital cerebrum was lined by reactive astrocytes, hemosiderin-laden macrophages and rare lymphocytes. The catheter bed in the ventricle exhibited atrophic ependymal cells.

**Table 2:** Ventricular dilatation and outcome in hydrocephalic sheep with shunt treatment

**Sheep #**	**Post kaolin day shunted**	**Post kaolin day of sacrifice**	Ventricle enlargement Ratio V/C*	**Comments**
Controls			None	Non-treated normal animals
			(0.030)	
			None	
			(0.028)	
77R	7	16	Moderate	Found dead; shunt failure - choroid plexus ingrowth
			(0.180)	
78R	7	15	Moderate	Shunt failure - choroid plexus ingrowth / hemorrhage
			(0.129)	
194R	7	63	Moderate	Shunt failure - choroid plexus ingrowth
			(0.113)	
162R#	7	126	Mild	Shunt tip in choroid plexus / occlusion
			(0.089)	
102S	6	6		Problems with shunt insertion
113S	6	6		Intraoperative hemorrhage
118S	6	8	Mild	Meningitis and ventriculitis; catheter occluded by inflammatory cells
			(ND)**	
28S#	6	84	Mild	Shunt failure - choroid plexus ingrowth
			(0.059)	
75S	7	10	Mild	Asymmetric enlargement of ventricles, blood in CSF, ventriculitis, catheter occluded by inflammatory cells and blood
			(0.053)	
115S#	7	84	Moderate	Shunt failure - choroid plexus ingrowth
			(0.152)	
5S	7	11	Mild	Shunt failure - misplacement of catheter
			(0.062)	
104S	7	9	Moderate	Intraventricular / intraparenchymal hemorrhage; ventricular catheter unobstructed
			(0.115)	
129S	8	17	Mild	Shunt patent
			(0.077)	
0379	8	13	Mild	Ventriculitis, meningitis; shunt occluded by hemorrhagic material and bacterial debris
			(0.074)	
7023#	8	63	Mild	Shunt patent
			(0.063)	
47#	14	77	None 0.024)	Brain tissue growth into shunt

# - animals that did well after shunting and were sacrificed at time rather than for clinical considerations. * V/C – ratio ventricle area/cerebrum area. **ND – not done.

The proximal catheter often contained choroid plexus (example in Figure 3A) with microscopic evidence of focal hemorrhage, fibrosis and reactive giant cells. The outside of the catheter was wrapped by collagenous membrane with some hemosiderin containing macrophages and lymphocytes. Despite antibiotic prophylaxis, two sheep had bacterial infection and the shunt catheter was occluded by inflammation.

## Discussion

In this study, our objective was to implant shunts in sheep with hydrocephalus and obtain insights into shunt function/failure. In this regard, mild to moderate ventricular expansion was produced consistently with 3 ml of a 25% kaolin injection. Kaolin was chosen for this model since it has been used extensively to induce hydrocephalus in a variety of animals including rats [14], rabbits [15], hamsters [16], cats [17], dogs [18,19] and fetal sheep and monkeys [12]. The ventriculomegaly produced by introduction of this agent into the cisterna magna is thought to be largely obstructive in nature primarily due to blockage of the fourth ventricle outlets. The inflammation and fibrosis mimics that which follows meningitis and subarachnoid hemorrhage [20]. It should be noted that the lymphocyte infiltration was considerably greater than seen previously in rodents [21], cats [22] or ferrets [23]. The inflammation and fibrosis in the subarachnoid compartment is most severe around the brainstem and cervical spinal cord; this is the likely cause of the pressure discontinuity between the brain and spinal subarachnoid compartments, but it did not generally result in enlargement of the central canal of the spinal cord. Kaolin-induced hydrocephalus in adult sheep appeared to be very similar to that in other species; in general severe ventricular enlargement is seen only when hydrocephalus is induced in young animals. The relatively mild ventricle enlargement was associated with only minor reactive glial changes in the periventricular white matter and no significant tissue destruction. This is typical also [24].

We used sheep because of the size and placid disposition. The size of the brain allowed us to use shunts that were very similar in design and size to their human counterparts. With the enlarged ventricles, shunting was relatively easy in sheep and intraoperative problems with shunt placement affected only a limited number of animals. We can say generally, that the animals deteriorated after kaolin injection and demonstrated a mixed response following shunting. Some sheep remained stable until sacrifice (31%) although we must interpret these ‘successes’ cautiously since imaging was not performed prior to shunt insertion and consequently, the change in ventricular size cannot be determined. In the remaining 69%, the clinical condition after shunting eventually worsened and they were euthanized. In 11 of the shunted animals, one could make the case that shunt failure contributed to the poor clinical state and in these sheep, euthanasia was required. However, the relationship between shunt status and clinical condition was not always clear. Three clinically stable sheep had shunts that appeared to be blocked with choroid plexus. Therefore, it appeared that some animals improved whether or not the shunt was functional although it is possible that shunt failure had occurred relatively close to the time of sacrifice and that deterioration of the clinical state would have been observed a short time after termination of the experiment. In general, the deterioration of clinical status seems to be related more to elevation of intracranial pressure than to distortion of the brain by ventriculomegaly.

The patency of the holes in ventricular shunts is essential for normal function. Apart from the occasional misplacement, shunt malfunction in sheep appeared to be due to the blockage of the holes with choroid plexus, brain tissue, or hemorrhagic and inflammatory debris. In some cases, the outsides of the proximal catheters were wrapped by a collagenous membrane with foreign body-type reactive giant cells. These findings mirror those obtained in the clinical setting [25].

Fetal and neonatal sheep have been useful for hydrocephalus studies [12,26-28]. In some experiments the hydrocephalus has been treated with ventricular-amniotic, ventricular-atrial or ventricular-pleural shunts. *In utero* shunting of fetal sheep with kaolin-induced hydrocephalus improved mortality and ventricle size [29-31].

The animal models noted earlier have all contributed to our understanding of the pathophysiology of hydrocephalus and each no doubt, has its place in the field. However, there would appear to be some merit in developing larger animal models in which the anatomical relationships and shunt sizes are more similar to those used in humans. The use of dogs would be an obvious choice. As was the case we observed in sheep, kaolin administration to dogs represents a serious challenge to the animals. In one study in which 50 mg/kg kaolin was administered into the cisterna magna, the mortality rate was 38% [32]. In any event, the negative publicity associated with the use of dogs in research in some locations discourages their use. Similarly, ethical issues and costs limit the appeal of utilizing non-human primates for hydrocephalus studies. In contrast, the use of adult sheep has some advantages. Sheep costs are relatively low and availability is not usually a problem. CSF physiology in this species has been studied extensively [9,10,33,34]. These animals generally adapt to surgical procedures well and are relatively easy to maintain. Furthermore, post-mortem analysis suggested that the shunts failed in ways that were very similar to their human counterparts. It would appear then, that this model replicates the type of shunt failure in humans and as such, can provide a foundation on which to build and test new shunt designs that negate the more unfortunate host-shunt interactions that occur in the clinical setting.

It must be noted however, that there are some disadvantages to use of sheep. This model is very labor intensive with a requirement of constant care after kaolin administration and timely interventions at frequent intervals. Furthermore, the relative short-term survival of the sheep after shunting may not replicate the longer-term host-shunt interactions that will no doubt be operative in the clinical setting. In other animal models shunts have remained patent for relatively long periods. In experiments using neonatal cats, ventricular catheters were functional for up to 12 weeks [35]. The cats had initial ventriculomegaly much greater than the sheep in our study and the cats’ ventricles remained large despite frequent intermittent CSF drainage. This example underscores the importance of measurements of initial ventricle size and highlights one of the disadvantages of sheep use for hydrocephalus studies. In some institutions it is very difficult to image sheep ventricles with MRI due to restrictions related to the risk of Q-fever transmission (*Coxiella burnetii*). Indeed, in some institutions, sheep use is prohibited entirely for this reason. Additionally, it is possible that mild to moderate ventricular expansion is all that can be achieved in adult animals.

## Conclusions

The injection of kaolin into the cisterna magna of adult sheep provides a useful model of hydrocephalus. This species is amenable to shunting with anatomical relationships and shunts that are similar to their human counterparts. The shunts fail in ways that are highly reminiscent of the neurosurgical experience with patients. Therefore, this animal model may be helpful in the development of new shunt technologies that enhance the effectiveness of CSF diversion.

## Competing interests

The authors (MGJ, JMD, DA, DDC) declare that they have no competing interests. MDB was a paid consultant for the study and JB is an employee of Medtronic Neurosurgery.

## Authors’ contributions

MGJ: conceived of the study and participated in its design and coordination. MDB: performed the histopathological assessment of the brains; participated in the study design and coordination. JMD: performed the surgical procedures and participated in the study design and coordination. DA: aided in the surgical procedures and maintenance of the sheep. DLD assessed the ventricle sizes and did the immunohistochemical studies. JB: conceived of the study; and participated in the study design and coordination. All authors have read and approved the final version of the manuscript.
